# Effect of omega-three supplementation on serum urate and gout flares in people with gout; a pilot randomized trial

**DOI:** 10.1186/s41927-022-00263-1

**Published:** 2022-06-08

**Authors:** Lisa K. Stamp, Rebecca Grainger, Christopher Frampton, Jill Drake, Catherine L. Hill

**Affiliations:** 1 Department of Medicine, University of Otago, Christchurch, PO Box 4345, Christchurch, 8140 New Zealand; 2 University of Otago, Wellington, Wellington, New Zealand; 3 University of Adelaide, Adelaide, South Australia; 4 The Queen Elizabeth Hospital, Adelaide, South Australia

**Keywords:** Gout, Omega-three supplementation, Clinical trial, Serum urate

## Abstract

**Objectives:**

To determine the effect of omega-three supplementation with fish oil on serum urate, weight and body mass index (BMI) in people with gout.

**Methods:**

A pilot 6-month, randomized, open-label clinical trial was undertaken in people with gout with serum urate ≥ 0.36 mmol/l. Forty participants were randomized to receive 6.2 g omega-3 fish oil daily or no fish oil for 24 weeks. Blood was obtained monthly for serum urate and red cell EPA (20:5n-3) DHA (22:6n-3) were measured using a blood spot collection system.

**Results:**

There was no statistically significant difference in the mean (SEM) decrease in serum urate between baseline and week 24 between randomized groups: fish oil − 0.021 (0.02) mmol/l versus control − 0.006 (0.02) mmol/l. There was no significant difference in change in weight or BMI between baseline and week 24 between randomized groups. There was a statistically significant correlation between red cell omega-three concentrations and the total number of flares per participant between week 12 and week 24; total omega-three r =  − 0.75 (*p* ≤ 0.001), EPA r =  − 0.75 (*p* ≤ 0.001) and DHA r = -0.76 (*p* ≤ 0.001). In the omega-three fish oil group four participants reported gastrointestinal adverse effects definitely or probably related to the omega-three supplementation.

**Conclusions:**

The lack of untoward effect of omega three fish oil supplementation on serum urate and BMI together with the relationship between higher omega-three concentrations and lower gout flares supports the development of further adequately powered clinical trials to determine the role of omega-three supplements as prophylaxis against gout flares in people starting urate lowering therapy.

*Clinical trial registration* ACTRN12617000539336p Registered 13/04/2017.

## Introduction

There are two primary goals in the management of gout. Firstly, gout flares must be treated to rapidly resolve the pain and swelling due to the inflammation caused by monosodium urate crystals (MSU) crystals. Secondly, long-term urate lowering therapy such as allopurinol is required to reduce serum urate which will ultimately lead to dissolution of MSU crystals and cessation of gout flares and tophi. Commencement of urate lowering therapy can be associated with an increase in gout flares [1, 2]. It is therefore recommended that patients receive prophylaxis against gout flares for the first 3–6 months of urate lowering therapy [3]. Colchicine or non-steroidal anti-inflammatories (NSAIDs) are recommended with low dose corticosteroid reserved for people in whom these agents are contra-indicated or ineffective [3]. Importantly NSAIDs and colchicine can have significant adverse effects and there are several important drug interactions. They are both relatively contra-indicated in the elderly and those with renal impairment, which limits their use for many people with gout. Furthermore, even low dose corticosteroids for six months can be associated with adverse effects. Thus, the current therapeutic strategies for prophylaxis against gout flares during initiation of urate lowering therapy are not appropriate or tolerable for many people with gout.

NSAIDs are non-selective, reversible inhibitors of cyclooxygenase (COX), the enzyme which catalyzes the synthesis of inflammatory prostaglandins and thromboxane from arachidonic acid (AA). Eicosapentaenoic acid (EPA), and docosahexaenoic acid (DHA), also known as omega-3 fats, must be consumed in the diet and thus are essential fatty acids. Omega-3 fats are found in high concentration in the oil derived from the tissues of cold-water fish (fish oil) and in plant-based foods such as linseeds. EPA and DHA act as alternate COX substrates to AA and their metabolism results in production of prostaglandins which are less inflammatory than those derived from AA. Thus omega-3 fatty acids can act as a “natural” anti-inflammatory drug.

Studies in people with rheumatoid arthritis show that regular consumption of anti-inflammatory doses of omega-3 fats (~ 3 g daily for most adults) results in reduction of use of NSAIDs. To date there have been no clinical studies of the effects and role of omega-3 fatty acids in people with gout. However, given their anti-inflammatory effects and favorable adverse effect profile there is a strong rationale for the use of omega-3 fatty acids in gout. Furthermore, a recent case–control study reported that high omega-3 fatty acid levels were associated with fewer gout attacks [4]. Therefore regular use of anti-inflammatory doses of omega 3 fish oil may be particularly useful as a prophylaxis against gout flares during the introduction of urate lowering therapy, and acceptable to patients due to tolerability and lack of major adverse effects compared to other prophylactic medications.

Omega-3 fatty acids have a number of advantages over the other medicines currently generally used for prophylaxis when starting urate lowering therapy. Importantly they do not have the severe gastrointestinal or potential cardiovascular adverse effects associated with colchicine or NSAIDs, nor is their use limited by kidney function. However, an important potential adverse effect is weight gain. In a recent two-year study of high (4.5 g daily) vs. low dose (0.45 gm daily) omega-3 supplementation in people with knee osteoarthritis, there was a mean (SD) weight increase of 1.7 (0.3) kg in the high dose and 0.6 (0.3) kg in the low dose group [5]. Therefore, it is possible that supplementation with omega-3 fatty acids may increase weight and BMI, an unacceptable adverse event in a patient population already burdened with high prevalence of obesity. Sustained reduction in serum urate to < 0.36 mmol/l is the key goal of long-term gout management and any treatment that acted to increase serum urate would be unadvisable.

Before omega-3 fatty acid supplementation can be routinely recommended for the management of gout further data, particularly on efficacy, is required. However, before a full clinical trial can be undertaken adherence to omega 3 supplementation must be confirmed and effects on serum urate and weight understood. The aims of this pilot study were to determine the effect of omega three fish oil supplementation on serum urate and weight/BMI in people with gout.

## Methods

### Study design

A pilot 6-month, randomized, open-label clinical trial was undertaken (ACTRN12617000539336p). Participants were enrolled between July 2014 and November 2018. Ethical approval was obtained from the New Zealand Health and Disability Ethics committee (17/NTB/68) and all participants provided written informed consent. All study procedures were carried out in accordance with relevant guidelines and regulations.

### Participants

People ≥ 18 years of age with gout as defined by the 2015 Gout Classification Criteria [6] with serum urate ≥ 0.36 mmol/l and either on a stable dose of allopurinol for at least one month or on no urate lowering therapy were recruited. People with a history of intolerance or allergy to omega three fatty acid supplements or an allergy to fish or shellfish were excluded as were people with an implantable defibrillator or receiving warfarin or dabigatran.

### Randomization and masking

The randomization sequence was generated electronically by an independent statistician. The randomization sequence was stratified by allopurinol use and arranged in permuted blocks of size four. Participants were randomized on a 1:1 ratio to receive omega-3 fatty acid supplementation or not (controls). Randomization codes were provided to the study coordinator in a sealed opaque envelope which was opened after the participant had consented.

### Study treatment and procedures

Those participants randomized to receive omega 3 fish oil were provided with commercial fish oil manufactured by Melrose (Mt Waverley, Victoria, Australia) at baseline and week 12 (i.e. three-month supply) with instruction to store per the manufacturer’s recommendation. Participants were instructed to take 20 ml daily to provide a total of 6.2 g omega-3 fatty acids. Melrose fish oil was chosen as it is easily available, has been used in other recent clinical trials of omega-three supplementation in both osteoarthritis and rheumatoid arthritis in Australia [6]. The control group received no intervention. For those on allopurinol the dose remained stable during the entire study period.

Participants were seen at baseline, week 12, and week 24 by the study coordinator with monthly telephone assessment. At each assessment, concomitant medications, self-reported gout flares requiring treatment, and adverse events were recorded. Blood was obtained monthly for serum urate and creatinine and three-monthly for full blood count and liver function tests. HBA1c, cholesterol, HDL, and LDL concentrations were measured at baseline and week 24. For people on allopurinol oxypurinol was also measured at baseline and week 24. Adherence was assessed at each in-person study visit by volume of fish oil remaining and red cell EPA and DHA concentrations. Red cell EPA and DHA were measured using a blood spot collection system as previously described [7, 8]. Samples were stored and analyzed in two batches in Adelaide, Australia.

### Adverse and serious advent event reporting

Adverse events (AEs) and serious adverse events (SAEs) were coded according to Common Terminology Criteria for Adverse Events (CTCAE v4.0). Participants were asked about occurrence of any AEs as well as specific fish oil related AEs (abdominal pain, nausea, bloating, reflux). Treatment emergent AEs were defined as any AE occurring after entry into the study until the end of week 28. Worsening laboratory AEs were defined as those where there was an increase in CTCAE grade between baseline and week 28. Serious adverse events were defined as an event that was life-threatening, required hospital admission or resulted in death. AEs and SAEs adverse events were classified as not related, possibly, probably, or definitely related to fish oil. Management of AEs was at the discretion of the treating physician.

### Study outcomes

The primary efficacy outcome was the change in serum urate between baseline and week 24 (or the final visit for those who discontinued fish oil or were lost to follow-up). Secondary outcomes included (1) change in weight and BMI between baseline and week 24, (2) mean number of flares per month between 0 and week 24, (3) percentage of participants with flares at each month, (4) cessation of fish oil therapy prior to week 24 due to adverse effects or intolerance and (5) change in HBA1c, cholesterol, HDL, and LDL concentrations between baseline and week 24 and in oxypurinol for people on allopurinol.

### Sample size and power

This pilot study was designed to answer our basic aims which will allow us to design a large clinical trial examining the role of omega-3 fatty acids in gout prophylaxis. The sample size of 20 per group was not based on formal statistical considerations but to allow sufficient information meet aims and, if appropriate, to inform design of an appropriately powered clinical trial.

### Statistical analysis

Baseline demographics and clinical features were summarized using descriptive statistics including means, standard deviation (SD), median, range, number and percent as appropriate. All randomized participants were included within the intention-to-treat (ITT) analysis population and analyzed within their randomized group. The primary efficacy outcome, change in serum urate, was compared between randomized groups using a general linear model which included randomized group and allopurinol use as fixed factors and baseline serum urate as a co-variate. The analyses of continuous secondary outcomes were undertaken using the same statistical model as for the primary outcome with the appropriate baseline variable as the covariate and the mean differences in the changes summarized with 95% confidence intervals. The percentage of participants with at least one gout flare were compared between randomized groups using Chi square or Fisher’s exact test as appropriate. The associations between the changes in total omega three, EPA and DHA and change in serum urate were tested using Pearson’s correlation coefficient. Correlations between omega three fatty acid concentrations and gout flares were tested using Spearman’s correlation coefficient. A two-tailed p value < 0.05 was taken to indicate statistical significance. The variables, analysed using parametric methods, were adequately normally distributed and this were confirmed by visual inspection of the residual plots from the general linear models. All analyses were undertaken using SPSSv27.

## Results

### Participant characteristics

**Fig. 1:**
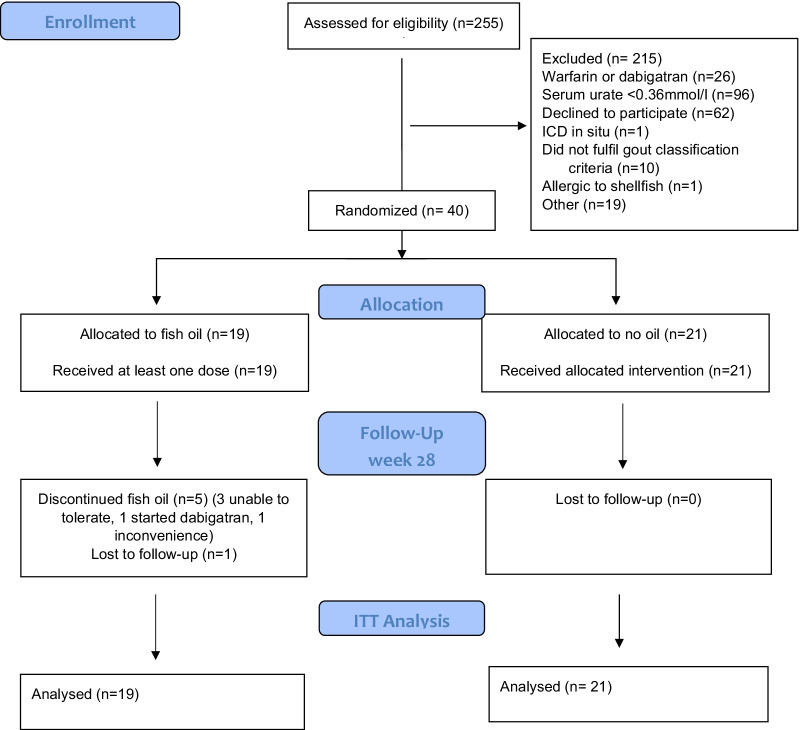
CONSORT flow of participants

Of the 255 individuals screened, 40 were randomly assigned to receive omega 3 fish oil (n = 19) or no fish oil (n = 21) (Fig. 1). All participants randomized to the fish oil group received at least one dose and no participants in the no fish oil group commenced it outside the trial setting. Five participants in the fish oil group discontinued the oil; three were unable to tolerate the taste, one commenced dabigatran and one found it inconvenient to take it (Fig. 1). The baseline demographic and clinical features were well matched between randomized groups (Table 1).

**Table 1:** Participant demographics, comorbidities and laboratory values

	Omega 3 (n = 19)	Control (n = 21)	Total (n = 40)
Male	14 (73.7%)	20 (95.2%)	34 (85.0%)
NZ European	13 (68.4%)	18 (85.7%)	31 (77.5%)
Age years)	63.3 (12.3)	59.3 (14.7)	61.2 (13.6)
Weight kg	89.0 (22.3)	91.4 (19.2)	88.8 (20.6)
BMI kg/m^2^	30.3 (6.5)	29.7 (5.2)	30.0 (5.8)
Hypertension	7 (36.8%)	9 (42.9%)	16 (40%)
Hyperlipidaemia	5 (26.3%)	5 (23.8%)	10 (25%)
Type 2 diabetes	3 (15.8%)	1 (4.8%)	5 (10%)
Ischaemic heart disease	0 (0)	2 (9.5%)	2 (5%)
Serum urate mmol/l	0.44 (0.08)	0.46 (0.08)	0.45 (0.08)
eGFR (ml/min/1.73m^2^)	68.5 (15.6)	69.7 (18.9)	69.1 (17.2)
Cholesterol (mmol/l)	5.4 (1.0)	5.2 (1.1)	5.3 (1.0)
Triglycerides (mmol/l)	2.1 (0.8)	2.4 (1.3)	2.3 (1.1)
HDL (mmol/l)	1.3 (0.3)	1.2 (0.3)	1.2 (0.3)
LDL (mmol/l)	3.1 (1.0)	3.0 (0.9)	3.0 (0.9)
Cholesterol (total/LDL)	4.3 (1.1)	4.5 (1.4)	4.4 (1.3)
Total red cell omega 3%	4.6 (1.2)	4.4 (0.9)	4.5 (1.0)
20:5n-3 (EPA)%	0.8 (0.3)	0.8 (0.3)	0.8 (0.3)
22:6n-3 (DHA)%	2.1 (0.5)	1.9 (0.4)	2.0 (0.5)
HbA1C (mmol/mol)	41.4 (16.2)	35.8 (11.1)	38.5 (13.9)
On allopurinol	8 (42.0%)	9 (43.0%)	17 (42.5%)
Serum Oxypurinol (µmol/l)	69.3 (38.4) (n = 8)	42.8 (28.6) (n = 9)	55.3 (35.2)
Tophi present	4 (21.1%)	3 (14.3)	7 (17.5%)
Flare prophylaxis	4 (21.1%)	2 (9.5%)	6 (15%)

Data are presented as mean (SD) or n(%) unless otherwise stated

*NZ* New Zealand, *BMI* body mass index, *kg* kilogram, *eGFR* estimated glomerular filtration rate, *HDL* high density lipoprotein, *LDL* low density lipoprotein, *EPA* eicosapentaenoic acid and DHA docosahexaenoic acid

### Primary endpoint

**Fig. 2:**
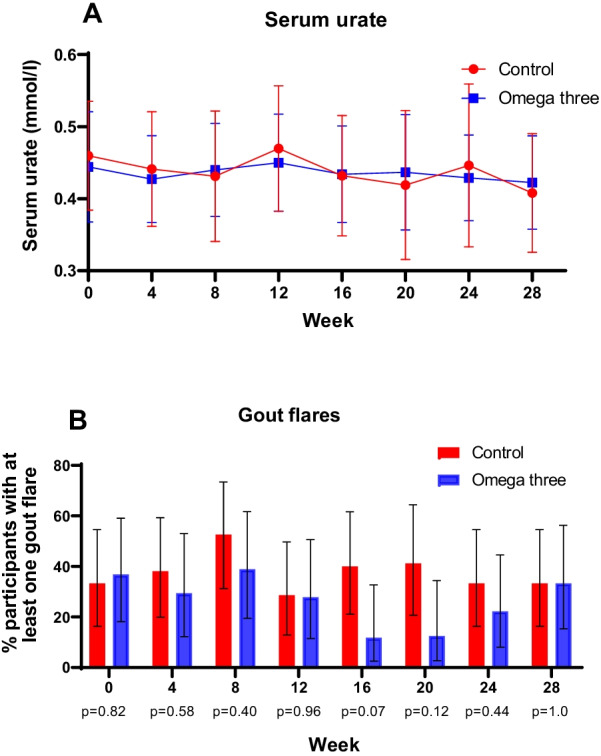
**A** Serum urate and **B** percentage of participants with at least one flare in the preceding four weeks over the study period in controls and those receiving omega three fish oil

There was no statistically significant difference in the mean (SEM) decrease in serum urate between baseline and week 24 between randomized groups: fish oil − 0.021 (0.02) mmol/l versus control − 0.006 (0.02) mmol/l with a mean difference between randomized groups of 0.015 (95% CI − 0.07 to 0.04) mmol/l (*p* = 0.59). There was no statistically significant difference in mean (SEM) serum urate at any time point over the study period (Fig. 2A).

### Gout flares

There was no statistically significant difference in the percentage of participants experiencing at least one flare each month (Fig. 2B). There was no significant difference in the percentage of participants having at least one flare per month from week 12–24 between randomized groups (*p* = 0.33). There was no statistically significant difference in the median (range) number of flares per participant from baseline to week 24 between groups; controls 2 (0–15) versus fish oil 1.5 (0–5) *p* = 0.34. Likewise, there was no statistically significant difference in the median (range) number of flares per participant from week 12 to week 24 between groups; controls 0 (0–12) versus fish oil 0 (0–3) *p* = 0.27. There was no significant difference in serum urate concentrations at week 24 and the occurrence of gout flares from week 12–24 (*p* = 0.90).

### Other secondary endpoints

There was no statistically significant difference in change in weight or BMI between baseline and week 24 between randomized groups (Table 2). Likewise, there was no difference in change in eGFR, cholesterol, HDL triglycerides, or HbA1C or plasma oxypurinol for those on allopurinol (Table 2). There was a statistically significant difference between change in LDL with those receiving fish oil having an increase in LDL and those with no oil having a reduction in LDL [mean (SEM) difference in change 0.36 (0.15) *p* = 0.02].

**Table 2:** Change between baseline and week 24 for secondary outcomes

	Omega three	Control	Mean difference (Omega three minus Control)	*p*
Weight (kg)	0.23 (0.49)	− 0.05 (0.46)	0.28 (− 1.09 to 1.65)	0.68
BMI (kg/m^2^)	− 0.01 (0.16)	0.03 (0.15)	− 0.04 (− 0.419 to 0.41)	0.86
eGFR (ml/min/1.73m^2^)	− 2.7 (1.5)	− 1.5 (1.4)	− 1.2 (− 5.3 to 2.9)	0.56
Cholesterol (mmol/l)	0.23 (0.13)	− 0.11 (0.12)	0.34 (− 0.03 to 0.71)	0.07
Triglycerides (mmol/l)	− 0.38 (0.18)	− 0.16 (0.16)	− 0.22 (− 0.71 to 0.27)	0.37
HDL (mmol/l)	0.07 (0.04)	0.02 (0.04)	0.05 (− 0.07 to 0.18)	0.39
LDL (mmol/l)	0.31 (0.11)	− 0.06 (0.10)	0.37 (0.07 to − 0.66)	0.02
Cholesterol (total/LDL)	0.06 (0.19)	− 0.09 (0.18)	0.15 (− 0.37 to 0.67)	0.57
HbA1c (mmol/mol)	1.08 (1.0)	0.77 (0.92)	1.85 (− 0.94 to 4.64)	0.19
Oxypurinol(µmol/l)	− 2.2 (10.9)	12.7 (9.7)	− 14.9 (− 47.3 to 17.4)	0.34
Total omega 3 (%)	5.69 (0.57)	0.38 (0.53)	5.31 (3.72 to 6.90)	< 0.001
20:5n-3 (EPA)%	2.90 (0.34)	− 0.04 (0.32)	2.93 (1.99 to 3.88)	< 0.001
22:6n-3 (DHA)%	2.15 (0.23)	0.14 (0.22)	2.01 (1.36 to 2.67)	< 0.001

Data presented are mean (SEM) and mean difference (95%CI), Week 24 minus Baseline

### Plasma EPA and DHA concentrations and flares

There was a statistically significant increase in total omega-3, EPA (c20:5n-3) and DHA (c22:6n-3) concentrations between baseline and week 24 in those who received fish oil which was not observed in controls (Table 2). There was no correlation between changes in omega three fatty acids and change in serum urate between baseline and month six: total omega-3 (r = 0.12, *p* = 0.49), EPA (c20:5n-3) (r = 0.15, *p* = 0.35) and DHA (c22:6n-3) (r = 0.15, *p* = 0.37). There was a statistically significant correlation between omega three concentrations and the total number of flares per participant between week 12 and week 24; total omega three r =  − 0.75 (*p* ≤ 0.001), 20:5n-3 (EPA) r =  − 0.75 (*p* ≤ 0.001) and 22:6n-3 (DHA) r =  − 0.76 (*p* ≤ 0.001). There were no flares between week 12 and 24 in those participants with total omega three > 8 (12 people in fish oil group), 20:n5 > 3 (11 in fish oil group) and 22:n6 > 3.5 (12 in fish oil group). None of the controls had fatty acid values above these cut offs.

### Adverse events

Adverse events are summarized in Table 3. Overall there were 65 adverse events in 15 participants receiving omega three fish oil supplement and 61 adverse in 17 controls. None of the 61 AEs in the control group were considered related. In the omega three fish oil group four participants reported nausea, dyspepsia and/or diarrhea definitely or probably related to the omega three supplementation. There were two serious adverse events, both in participants in the control group; a stroke and atrial fibrillation requiring hospital admission.

**Table 3:** Number of adverse events over the study period

	Omega 3 (n = 19)	Control (n = 21)	Total (n = 40)
Cardiac disorders	5	1	6
Ear and labyrinth	0	1	1
Eye disorders	0	1	1
Gastrointestinal	17	12	29
General disorders	7	7	14
Infections and infestations	5	8	13
Injury, poisoning and procedural complications	4	1	5
Investigations	1	0	1
Musculoskeletal	18	12	31
Neoplasm	0	1	1
Nervous system disorders	2	5	7
Psychiatric disorders	1	2	3
Renal and urinary	0	1	1
Respiratory	3	6	9
Skin and subcutaneous	1	1	2
Vascular disorders	1	1	2
Total	65	61	126

## Discussion

Approximately a quarter of people with gout take some form of complementary therapy [9]. While adherence with medication is generally poor in people with gout, many patients report a preference for dietary-based and complementary therapies. Many complementary therapies, including omega-3 fatty acid supplementation, are expensive and have little data to support their use. Herein we have shown that omega-3 fish oil supplementation has no significant effect on serum urate or BMI over a six-month study period.

There is limited data on the effect of omega three fatty acids on serum urate. An in vitro study using URAT1-expressing 293A cells reported that EPA and DHA were strong inhibitors of URAT1 dependent urate transport leading the authors to suggest these fatty acids could have urate lowering potential in humans through their uricosuric effects [10]. In a small study of 17 health men aged on average 60 years, consumption of 700 mg of omega three fatty acids (260 mg DHA and 260 mg EPA) and strength training for three months resulted in a statistically significant reduction in serum urate; pre median (range) 0.37 (0.27–0.63)mmol/l and post 0.36 (0.24–0.48) mmol/l; *p* = 0.017 [11]. In another small study of healthy young people consumption of 2 g fish oil daily (each 1 g capsule containing 490 mg DHA and 98 mg EPA) for eight weeks led to a significant reduction in serum urate; mean (SD) baseline 0.347 (0.025) mmol/l; week four 0.260 (0.017) mmol/l and week eight 0.308 (0.018) mmol/l *p* < 0.05 [12]. In our study we observed no effect of omega three fish oil supplementation on serum urate. Importantly, our study was undertaken in people with gout and the most common cause of hyperuricamia in people with gout is renal under-excretion of urate [13]. Thus, while the uricsouric effects of EPA and DHA may be sufficient to lead to a reduction in serum urate in individuals with normal renal urate excretion it may not be enough in the setting of decreased renal urate excretion. Furthermore, the uricosuric effect of EPA and DHA may be diminished in the presence of renal impairment or concomitant therapy with diuretics and/or aspirin.

In our study there was no effect of omega three fish oil supplementation on gout flares although this study was not powered to detect differences in flares. Two other studies have reported the effects of omega three supplementation on gout flares. In a large internet-based study dietary n-3 fish consumption was associated with a lower risk of self-reported gout flare after adjustment for total purine intake, an effect not seen in self-directed consumption of n-3 fatty acid supplements [14]. A case–control study reported that high omega-3 fatty acid levels were associated with fewer gout attacks after adjustment for age, BMI, disease duration, serum urate tertiles, presence of tophi and use of urate lowering therapy adjusted OR 0.62 (95%CI 0.38–0.98; *p*_trend_ 0.043) [4]. In this study the mean (SD) omega three concentration was 0.41 (0.11) mmol/l. We also observed a significant correlation between omega three concentrations and the total number of flares per participant between week 12 and week 24 flares with no flares between week 12 and 24 in those participants with total omega three > 8 (12 in fish oil group), 20:n5 > 3 (11 in fish oil group) and 22:n6 > 3.5 (12 in fish oil group). These data support a role for omega three supplementation in preventing gout flares and however suggest a minimum therapeutic concentration may be required.

Despite evidence of weight gain in a previous study of omega three fish oil supplementation in people with osteoarthritis [5], in the current study there was no significant increase in weight or BMI in people who received omega three supplementation. This is especially important since obesity is a major issue in people with gout; data from NHANES 2007–2008 revealed 53% of people with gout were obese [15]. A number of studies also confirm the positive association between BMI and serum urate concentration in univariate and multivariate regression analyses [16, 17]. Maintaining a healthy weight is a one of the key lifestyle recommendations for gout [18] thus therapies that might increase weight should be avoided. We chose our primary outcomes as serum urate rather than a patient important outcome such as gout flares as this study was designed to answer important questions required for development of a large clinical trial.

The most common adverse effects were gastrointestinal related to the fish oil. While these adverse effects of omega-3 fatty acid supplementation are clearly neither organ nor life-threatening, they can frequently lead to non-adherence. In the current study 3/20 (15%) participants discontinued the fish oil due to gastrointestinal adverse effects. This is similar to the non-adherence rate of 20% in a recent two-year study of fish oil in osteoarthritis [5]. Adherence with gout therapies is particularly poor with a recent systematic review reporting adherence rates of 10–46% [19]. This is often attributed to poor gout education and the intermittent nature of gout. Ensuring adequate adherence to meet adequate red cell omega 3 concentrations will be a high priority in future clinical studies and a major consideration for use in the clinical setting.

There are several limitations to our study. We did not use a placebo oil in part because of our interest in the effects of the fish oil supplement on weight. Participants were advised to maintain their usual diet and we did not control for dietary consumption of omega three fatty acids although people randomized to the control arm were advised not to take omega three supplements. While gout flares were self-reported which could be subject to bias, our primary outcome measure was serum urate which bias not subject to recall bias. Finally, we did not collect information on known triggers for gout flares such as trauma and dietary triggers.

In summary we have shown no effect of omega three fatty acid supplementation with fish oil on serum urate, weight or BMI in people with gout. The relationship between higher omega-three concentrations and lower gout flares in people on stable doses of allopurinol supports the development of further adequately powered clinical trials to determine the role of omega three supplements as prophylaxis against gout flares in people starting urate lowering therapy.

## Data Availability

Data is available upon request from the corresponding author subject to ethical approval.
